# Population-level impact of adjuvant trastuzumab emtansine on the incidence of metastatic breast cancer: an epidemiological prediction model of women with HER2-positive early breast cancer and residual disease following neoadjuvant therapy

**DOI:** 10.1007/s12282-023-01514-w

**Published:** 2023-11-01

**Authors:** Mellissa Williamson, David J. Press, Svenn Alexander Hansen, Akanksha Tomar, Gurleen Singh Jhuti, Cedric Revil, Kaustubh Gururaj

**Affiliations:** 1 Genentech, Inc., 1 DNA Way, South San Francisco, CA USA; 2 Gilead Sciences, Inc., Foster City, CA USA; 3 F. Hoffmann-La Roche AG, Basel, Switzerland; 4 Merck Sharp and Dohme, Zurich, Switzerland; 5 ZS Associates International Inc., London, UK

**Keywords:** Breast cancer, HER2-positive, Trastuzumab emtansine, Epidemiology, Neoadjuvant

## Abstract

**Purpose:**

Treating early-stage breast cancer (eBC) may delay or prevent subsequent metastatic breast cancer (mBC). In the phase 3 KATHERINE study, women with human epidermal growth factor receptor 2 (HER2)-positive eBC with residual disease following neoadjuvant therapy containing trastuzumab and a taxane experienced 50% reductions in disease recurrence or death when treated with adjuvant trastuzumab emtansine (T-DM1) vs adjuvant trastuzumab. We predicted the population-level impact of adjuvant T-DM1 on mBC occurrence in five European countries (EU5) and Canada from 2021–2030.

**Methods:**

An epidemiological prediction model using data from national cancer registries, observational studies, and clinical trials was developed. Assuming 80% population-level uptake of adjuvant treatment, KATHERINE data were extrapolated prospectively to model projections. Robustness was evaluated in alternative scenarios.

**Results:**

We projected an eligible population of 116,335 women in Canada and the EU5 who may be diagnosed with HER2-positive eBC and have residual disease following neoadjuvant therapy from 2021–2030. In EU5, the cumulative number of women projected to experience relapsed mBC over the 10-year study period was 36,009 vs 27,143 under adjuvant trastuzumab vs T-DM1, a difference of 8,866 women, equivalent to 25% fewer cases with the use of adjuvant T-DM1 in EU5 countries from 2021–2030. Findings were similar for Canada.

**Conclusion:**

Our models predicted greater reductions in the occurrence of relapsed mBC with adjuvant T-DM1 vs trastuzumab in the indicated populations in EU5 and Canada. Introduction of T-DM1 has the potential to reduce population-level disease burden of HER2-positive mBC in the geographies studied.

**Supplementary Information:**

The online version contains supplementary material available at 10.1007/s12282-023-01514-w.

## Background

In the European Union, breast cancer (BC) is the most common cancer among women [1], with an estimated 355,457 new cases and 91,826 deaths in 2020 [2] projected to increase to 368,524 new cases and 97,199 deaths in 2025 [3]. In Canada, BC is the second most common cancer overall, with an estimated 28,900 new cases and 5,500 deaths in 2022 [4]. Patients are most often diagnosed with stage I–III disease [5, 6], and demographic aging is projected to contribute to increases in new BC cases over time [1, 7, 8]. Prognosis correlates with stage at initial diagnosis and adherence to a standard of care for neoadjuvant and adjuvant treatment [5, 9]. Emerging evidence suggests that therapeutic advances are associated with population-level improvements in disease burden [8]. Moreover, treating early-stage BC (eBC)—when it is still potentially curable—may delay or prevent the recurrence of metastatic BC (mBC), easing population-level disease burden [8].

Given the acceleration of therapeutic advances in BC treatment in the last decade [10], alongside increases in BC disease burden, we expect that the population-level impact of BC treatments will accelerate over the next decade. Importantly, to our knowledge, no previous studies have predicted the future population-level impact of specific eBC treatments on the number of women who avoid metastases in Europe and Canada. Such a prediction model may help inform future clinical and financial support resources.

Human epidermal growth factor receptor 2 (HER2) is a biomarker of aggressive BC that is overexpressed in 25–30% of BCs [11]. The European Society for Medical Oncology recommends chemotherapy (i.e. docetaxel) plus trastuzumab and pertuzumab for neoadjuvant treatment of patients with HER2-positive BC, regardless of hormone receptor status [10]. However, approximately 40–60% of patients with HER2-positive BC fail to have a pathological complete response (pCR) following neoadjuvant treatment with this treatment regimen [12–14]. Compared with patients who have a pCR, the risk of disease recurrence or death is greater among those with residual disease following neoadjuvant therapy [15, 16].

The antibody–drug conjugate trastuzumab emtansine (T-DM1) was first approved in the European Union and Canada for the management of patients with HER2-positive mBC previously treated with trastuzumab and a taxane (separately or in combination) [17, 18]. In late 2019, both the European Medicines Agency and Health Canada approved adjuvant T-DM1 in patients with eBC and residual invasive disease following neoadjuvant chemotherapy plus HER2-targeted therapy [17, 18]. These approvals were based on the results of the phase 3 KATHERINE study, which found that adjuvant T-DM1 reduced the risk of invasive disease recurrence or death by 50% relative to adjuvant trastuzumab in patients with HER2-positive eBC and residual disease who were previously treated with neoadjuvant therapy containing a taxane and trastuzumab (hazard ratio for invasive disease or death, 0.50; 95% confidence interval, 0.39 to 0.64; *P* < 0.001) [19]. In 2020, following approval, adjuvant use of T-DM1 for indicated women began in France, Germany, Italy, Spain, and the United Kingdom (EU5) and Canada. We developed an epidemiological prediction model for the EU5 and Canada to estimate the population-level impact of adjuvant T-DM1 on preventing relapsed mBC in women with HER2-positive eBC and residual disease following neoadjuvant therapy.

## Methods

**Fig. 1:**
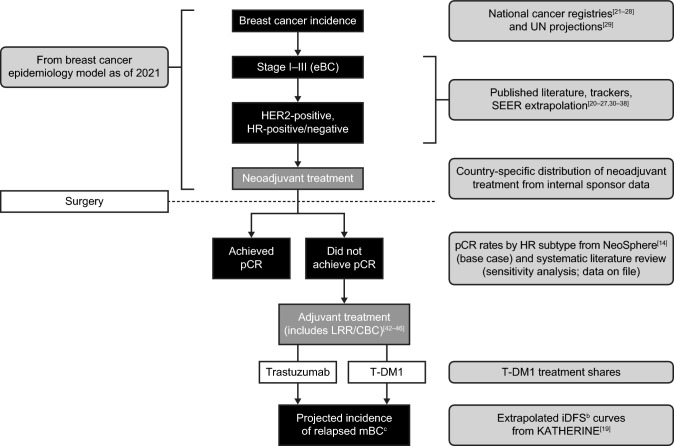
Patient flow and sources used to determine the numbers of prevented cases of relapsed mBC^a^, ^a^Over the 10-year study period, ^b^iDFS includes ipsilateral invasive breast tumor recurrence; ipsilateral locoregional invasive breast cancer recurrence; distant recurrence; contralateral invasive breast cancer; and death attributable to any cause, including breast cancer, non-breast cancer, or unknown cause, ^c^Relapsed mBC incidence includes patients with relapsed HER2-positive eBC, either direct relapse from adjuvant treatment or relapse from locoregional and contralateral recurrence. *CBC* contralateral breast cancer, *eBC* early breast cancer, *HER2* human epidermal growth factor receptor 2, *HR* hormone receptor, *iDFS* invasive disease-free survival, *LRR* locoregional recurrence, *mBC* metastatic breast cancer, *pCR* pathological complete response, *SEER* surveillance, epidemiology, and end results, *T-DM1* trastuzumab emtansine

To model the forecasted number of women diagnosed with HER2-positive eBC with residual disease following neoadjuvant therapy who may avoid relapse to mBC in the 10 years following European Medicines Agency (EMA) and Health Canada approvals of T-DM1 (2021–2030), we developed an epidemiological prediction model using data from observational and clinical trial studies, as summarized in Table 1 [10, 14, 19–41] and Fig. 1 [42–46]. We assumed 80% uptake of adjuvant T-DM1 among eligible women within 3 years following EMA and Health Canada approvals. The base models for EU5 and Canada assumed HER2-positive eBC was HR-positive in 70% and HR-negative in 30%. The number of women diagnosed with eBC was estimated based on historic incidence rate trends and applied to age-standardized incidence rates, as informed by country-specific cancer registries. Incidence projections were calculated based on the 5-year age-specific incidence rates using prospects in age structures from the United Nations [29]. Country-specific neoadjuvant treatment rates were then applied to the number of incident eBC diagnoses to estimate the frequency of women who received neoadjuvant treatment in each country. To estimate the numbers of women with residual disease following neoadjuvant treatment, we calculated weighted averages of pCR rates by HR status (HR-positive, 20%; HR-negative, 37%) from the NeoSphere trial [14]. Our models considered locoregional recurrence and contralateral breast cancer cases as receiving adjuvant treatment [42–46].

**Table 1:** Epidemiology model variables and sources used to determine the numbers of relapsed mBC cases

Variable	Definition	Source
Breast cancer incidence	Incident breast cancer diagnoses among women	National cancer registries for the EU5 [21–25] and Canada [26–28], and UN projections in age structures for future years [29]
eBC stage	Stage I–III	Published literature [20, 30–36], SEER data [37], and the EU5 [21–25] and Canadian [26, 27, 38] registries
HER2/HR status	HER2-positive, HR-positive, and HR-negative	Published literature [20, 30–36], SEER data [37], and the EU5 [21–25] and Canadian [26, 27, 38] registries Base models assumed HER2-positive eBC was HR-positive in 70% and HR-negative in 30%
HER2-positive neoadjuvant treatment rate	Rate of treatment with trastuzumab or trastuzumab plus pertuzumab	Country-specific data on file; treatment patterns associated with initial therapy as described by Byfield et al. [39]
HER2-positive, neoadjuvant-treated with residual disease (i.e. non-pCR)	Patients with HER2-positive residual disease following neoadjuvant treatment, stratified by neoadjuvant treatment regimens and HR subtype	NeoSphere trial [14] (base model); systematic literature review (data on file; pCR sensitivity analysis). All patients receiving neoadjuvant treatment were assumed to be treated with trastuzumab plus chemotherapy Base models assumed pCR rates of 20% in those with HR-positive disease and 37% in those with HR-negative disease Upper limit of the pCR sensitivity analysis assumed pCR rates of 43.8% in those with HR-positive disease and 73.2% in those with HR-negative disease
Relapsed mBC incidence	Extrapolated iDFS^a^ curves and pre-progression death rate	KATHERINE trial [19] using methods as described by Sussell et al. [40] and the Health Technology Assessment TA569 [41]

Over the 10-year study period

*eBC* early breast cancer, *ESMO* European society for medical oncology, *EU5* five European countries, specifically France, Germany, Italy, Spain, and the United Kingdom, *HER2* human epidermal growth factor receptor 2, *HR* hormone receptor, *iDFS* invasive disease-free survival, *mBC* metastatic breast cancer, *pCR* pathological complete response, *SEER* surveillance, epidemiology, and end results

^a^iDFS includes ipsilateral invasive breast tumor recurrence; ipsilateral locoregional invasive breast cancer recurrence; distant recurrence; contralateral invasive breast cancer; and death attributable to any cause, including breast cancer, non-breast cancer, or unknown cause

To estimate the yearly proportion of women with disease progression (locoregional or metastatic) or death, invasive disease-free survival (iDFS) curves from the KATHERINE trial [19] were extrapolated over a 10-year time horizon. The iDFS survival was extrapolated using a log-normal distribution, adjusted for cure proportion, duration of treatment effect, and background risk of death. Results and methodology used to extrapolate the iDFS data were the same as those presented by Sussell et al. [40] and are further described there. A similar methodology to extrapolate iDFS data in HER2-positive eBC has also been used in several models accepted by the National Institute of Health and Care Excellence [41, 47]. The best-fitting curves (log-normal) were used in the base case model, and iDFS curves were extrapolated for both the trastuzumab and T-DM1 treatment arms of KATHERINE. In the target population (women diagnosed with HER2-positive eBC with residual disease post-neoadjuvant therapy), the extrapolations from KATHERINE indicated improved iDFS with adjuvant T-DM1 vs adjuvant trastuzumab (Supplemental Fig. 2).

The resulting annual transition probability of being alive and progressing to mBC was then estimated by: calculating the yearly proportion of women with an event as informed by the 10-year extrapolated iDFS curves; removing the proportion of events expected to be death (without prior progression events), estimated at 2% for both arms, as informed by the KATHERINE trial; and splitting the remaining recurrences between non metastatic (local) and metastatic recurrences, for which it was assumed that 78.1% of recurrences would be metastatic, as informed by an observed period from the KATHERINE trial (196 out of 251 non-death iDFS events was observed to be metastatic) [19]. In the women with a local recurrence, the risk of having a second recurrence was based on Hamilton et al. [55]. From this, it was estimated that over a 10-year time period, 59.8% of women would have a subsequent metastatic recurrence [48].

Sensitivity analyses under different assumptions were performed to assess the magnitude of the change relative to the base model in the number of women projected to experience BC relapse following receipt of adjuvant T-DM1 vs. adjuvant trastuzumab (Table 2). Sensitivity analyses considered differences in treatment share, pCR rates, duration of treatment effect, and the impact of a simpler exponential model (i.e. constant hazard) on the iDFS extrapolation distribution (Supplemental Methods).

**Table 2:** Women projected to experience breast cancer relapse by geographic setting, adjuvant treatment regimen (T-DM1 or trastuzumab) and time period, among women diagnosed with HER2-positive eBC who have residual disease following neoadjuvant therapy from 2021 to 2030: sensitivity analyses

Analytical scenarios	Women projected to experience BC relapse from 2021 to 2030		
	Following trastuzumab (n)	Following T-DM1 (n)	Difference across scenarios
Five European countries (EU5)^a^			
Base model	36,009	27,143	(Reference)
(1) Assumed cure is not possible	44,283	34,763	6.9%
(2) Assumed the incremental treatment effect of T-DM1 over trastuzumab stops at 63 months (maximum iDFS follow-up from KATHERINE^b^)	36,009	28,357	− 15.9%
(3) Assumes iDFS extrapolation distribution is exponential (not log-normal)	40,334	30,975	5.3%
(4) Combines the above assumptions	52,715	45,769	− 27.6%
(5) Assumed high pCR rates from KRISTINE^c^	24,225	18,239	− 48.1%
(6) Assumed a peak 100% treatment proportion for T-DM1	36,009	24,926	20.0%
(7) Assumed a 25% relative increase in neoadjuvant treatment for all years	46,086	34,709	22.1%
(8) Assumed a 25% relative decrease in neoadjuvant treatment for all years	27,652	20,825	− 29.9%
Canada			
Base model	2,318	1,733	(Reference)
(1) Assumed cure is not possible	2,805	2,179	6.5%
(2) Assumed the incremental treatment effect of T-DM1 over trastuzumab stops at 63 months (maximum iDFS follow-up from KATHERINE^b^)	2,318	1,807	− 14.5%
(3) Assumes iDFS extrapolation distribution is exponential (not log-normal)	2,580	1,964	5.0%
(4) Combines the above assumptions	3,306	2,837	− 24.7%
(5) Assumes high pCR rates from KRISTINE^c^	1,466	1,096	− 58.1%
(6) Assumed a peak 100% treatment proportion for T-DM1	2,318	1,586	20.1%
(7) Assumed a 25% relative increase in neoadjuvant treatment for all years	2,897	2,166	20.0%
(8) Assumed a 25% relative decrease in neoadjuvant treatment for all years	1,738	1,299	− 33.3%
(9) Assumed a doubling of neoadjuvant treatment for years 2020 onwards	4,142	2,989	49.3%

*BC* breast cancer, *eBC* early-stage breast cancer, *EU5* five European countries, *HER2* human epidermal receptor growth factor 2, *HR* hormone receptor, *iDFS* invasive disease-free survival, *pCR* pathological complete response, *T-DM1* trastuzumab emtansine

^a^Includes Germany, Spain, Italy, the United Kingdom, and France

^b^KATHERINE ClinicalTrials.gov number, NCT01772472

^c^For the pCR rate sensitivity analysis, we conducted a systematic literature review to identify pCR rates following neoadjuvant treatment with trastuzumab in observational studies and also considered pCR rates from applicable randomized controlled studies, all of which were used in the sensitivity analysis. Here we report the upper limit of this sensitivity analysis, based on the highest pCR rates identified. These came from the docetaxel, carboplatin, and trastuzumab plus pertuzumab group of the KRISTINE study, which reported a pCR rate of 43.8% in those with HR-positive disease and 73.2% in those with HR-negative disease [49]. KRISTINE ClinicalTrials.gov number, NCT02131064

## Results

**Fig. 2:**
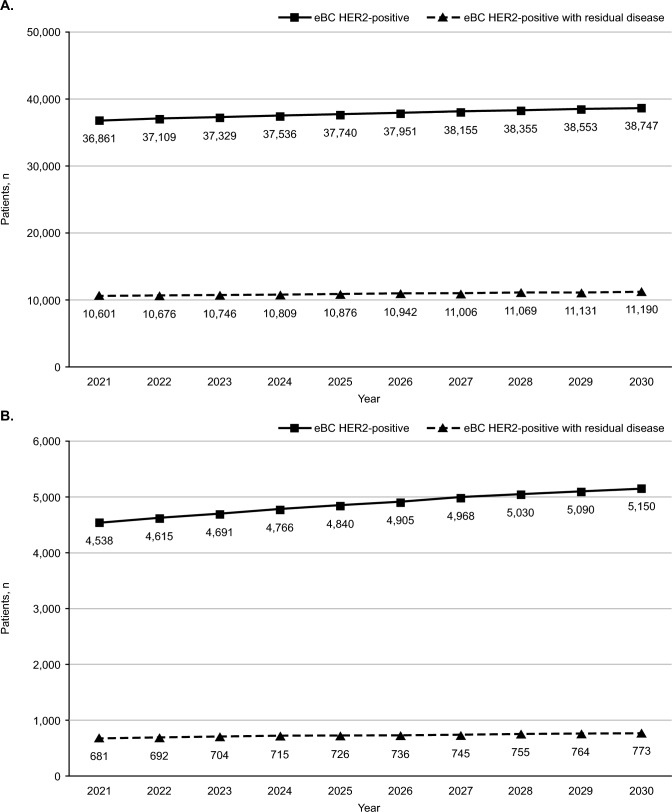
Women diagnosed with incident HER2-positive eBC from 2021 to 2030, overall and for those with residual disease following neoadjuvant therapy; in **A** the EU5 and **B** Canada. *eBC* early-stage breast cancer, *EU5* five European countries, specifically France, Germany, Italy, Spain, and the United Kingdom, *HER2* human epidermal growth factor receptor 2

Our models projected year-over-year increases in women diagnosed with HER2-positive eBC in both the EU5 and Canada over the next decade, with a total increase of 5% in the EU5 (from 36,861 in 2021 to 38,747 in 2030) and 12% in Canada (from 4,538 in 2021 to 5,150 in 2030) (Fig. 2). Our base models projected that a cumulative 378,336 and 48,594 women in EU5 and Canada, respectively, will be diagnosed with HER2-positive eBC from 2021–2030. Among those women, our models estimated that the proportion of women eligible for adjuvant T-DM1 (i.e. proportion diagnosed with HER2-positive eBC with residual disease following neoadjuvant therapy) would remain constant—29% in EU5 and 15% in Canada for all years. Moreover, our synthetic cohorts of women eligible for T-DM1 increased on a year-over-year basis in both geographies, with an estimated number of women followed for ≥ 1 year in EU5 of 109,045 and in Canada of 7,290, for a total of 116,335 eligible women.

**Fig. 3:**
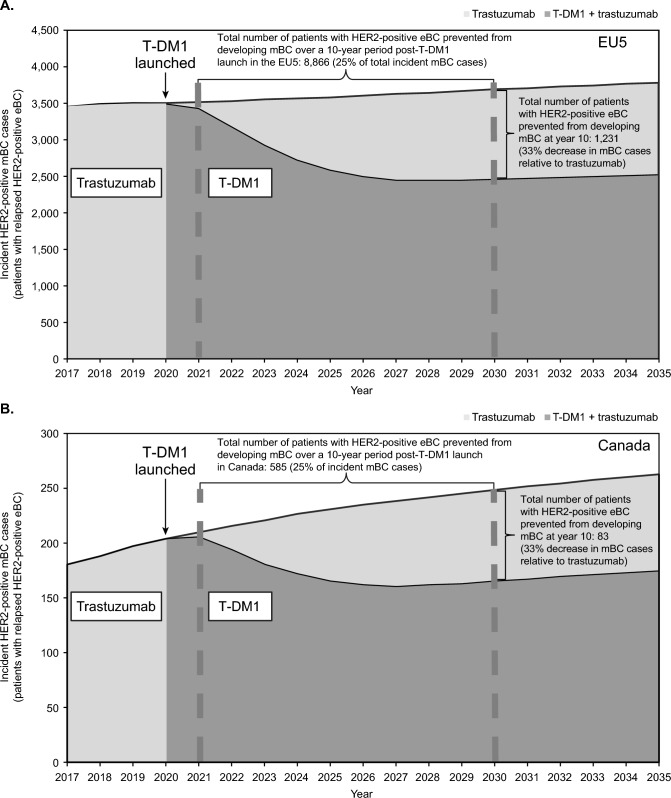
Projected women with HER2-positive eBC prevented from developing relapsed mBC in **A** the EU5 and **B** Canada. “T-DM1 + trastuzumab” assumes 100% uptake of T-DM1 in 3 years from the indication launch in 2020. *eBC* early breast cancer, *EU5* five European countries, specifically France, Germany, Italy, Spain, and the United Kingdom, *HER2* human epidermal growth factor receptor 2, *mBC* metastatic breast cancer, *T-DM1* trastuzumab emtansine

In the base model for EU5, we projected that the cumulative number of women experiencing relapsed mBC over the 10-year study period will be 36,009 under adjuvant treatment with trastuzumab and 27,143 under adjuvant treatment with T-DM1—a difference of 8,866 cases, which is equivalent to 25% fewer cases with T-DM1 (Fig. 3A). We projected that the annual number of women who will have avoided relapsed mBC with adjuvant T-DM1 vs adjuvant trastuzumab in EU5 would increase from 89 (2021) to 1,231 (2030). In 2030, the EU5 base model projected that the number of women experiencing relapse to mBC would be 3,691 under adjuvant trastuzumab and 2,460 under adjuvant T-DM1, corresponding to a 33% decrease with T-DM1. Findings were similar in analyses of individual EU5 countries (data not shown). In the base model for Canada, we projected that the cumulative number of women experiencing relapsed mBC over the 10-year study period will be 2,318 under adjuvant trastuzumab and 1,733 under adjuvant T-DM1—a difference of 585 cases, which is equivalent to 25% fewer cases with T-DM1 (Fig. 3B). The annual number of women who avoid relapse to mBC following treatment with adjuvant T-DM1 vs adjuvant trastuzumab in Canada is projected to increase from 5 (2021) to 83 (2030). In 2030, the base model projects 249 and 166 Canadian women relapsing to mBC following treatment with adjuvant trastuzumab and T-DM1, respectively, corresponding to a 33% decrease with T-DM1.

We present the results from our sensitivity analyses in Table 2 and Supplemental Table 1. The 10-year projected differences for both geographies were deemed to be unimportant (i.e. < 10%) between the base model and Scenarios 1 (assumes cure is not possible) and 3 (assumes iDFS extrapolation distribution is exponential [not log-normal]). In Scenario 2 we assumed that the T-DM1 incremental treatment effect over trastuzumab stops at 63 months (maximum iDFS follow-up from KATHERINE), and we observed a modest difference between the base model and Scenario 2 in both geographies. Specifically, the 10-year population-level benefit among women following treatment with T-DM1 vs trastuzumab decreased by 16% in EU5 (8,866 women in the base model and 7,652 women in Scenario 2) and by 15% in Canada (585 women in base model and 511 women in Scenario 2).

We observed a modest reduction in the percentage of women projected to experience mBC relapse over 10 years in Scenario 5 (assumed high pCR rates from KRISTINE [49]) vs the base model (assumed lower pCR rates from NeoSphere [14]). Specifically, we observed a smaller 10-year population-level benefit among women following treatment with T-DM1 vs trastuzumab of 48% in EU5 (8,866 women in base model and 5,986 women in Scenario 5) and 58% in Canada (585 women in base model and 370 women in Scenario 5). For the EU5, the sensitivity model using the high pCR rate estimates yielded the same percentage decrease in the total number of women with HER2-positive eBC prevented from developing relapsed mBC over the 10-year period compared with the base model. There were no proportional changes between the low vs high pCR rate models for the EU5 for the number of cases of relapsed mBC avoided at Year 10. Between the low vs high pCR rate models for Canada, there were also no proportional changes in the 10-year cumulative number of avoided mBC cases or the number of cases of relapsed mBC avoided at Year 10.

In Scenario 6 (assumed a peak 100% treatment proportion for T-DM1 vs. 80% in the base model), we observed cumulative increases in the benefit relative to the base model among women following treatment with T-DM1 vs trastuzumab of 20% in both geographies. Scenarios 7 through 9 demonstrated that results were highly sensitive to changes in neoadjuvant treatment uptake preceding eligibility for T-DM1. Specifically, a 25% relative increase in the administration of neoadjuvant treatment, as well as a doubling of neoadjuvant treatment in Canada, resulted in commensurate 10-year increases in the benefits of adjuvant T-DM1 vs trastuzumab relative to the base models. A 25% relative decrease in administration of neoadjuvant treatment resulted in decreases in the benefit of adjuvant T-DM1 vs trastuzuamb relative to the base models.

## Discussion

Treating BC at an early stage may delay or prevent progression to mBC, with recent improvements in HER2-targeted therapies optimized for women according to their risk of disease recurrence [9]. Reductions in disease progression following treatment improvements may translate to future population-level changes in disease burden. Using an epidemiological prediction model, we projected that the number of eligible women with HER2-positive eBC and residual disease following neoadjuvant therapy in the EU5 and Canada will increase between 2021 and 2030. Over the 10-year study period, we estimated that 25% fewer women would experience mBC under adjuvant T-DM1 vs adjuvant trastuzumab, and we projected that 8,866 women could avoid the occurrence of mBC with adjuvant T-DM1. Long-term projected increases in estimated patient benefit with adjuvant T-DM1 vs adjuvant trastuzumab were driven by the relative treatment benefits accruing to patients beyond the calendar year of T-DM1 treatment (i.e. accruing separation of survival curves evidenced in clinical trials).

We observed differences between the EU5 and Canada in the annual percentage change of women diagnosed with incident HER2-positive eBC with residual disease, which drove annual percentage change differences in our results. Variations were due to a larger annual percentage change in the number of women diagnosed with incident eBC in Canada vs EU5. These differences were more pronounced in Scenario 9 for Canada, which accounted for potential increases in neoadjuvant treatment before adjuvant T-DM1 eligibility following clinical changes around 2020. Altogether, these results suggest that the total number of patients with residual disease in Canada will increase over the next decade. This overall increase in patients eligible for adjuvant continuation treatment helps explain the increase we observed in metastatic recurrences in Canada for both the T-DM1 and trastuzumab arms of the model, a trend not observed from the EU5 results. These observed differences between EU5 and Canada may have implications for the healthcare systems in these regions, with a potential increased BC healthcare burden and need for additional services in Canada over the next decade. Recent Health Canada approval of pertuzumab in combination with trastuzumab and chemotherapy as neoadjuvant treatment of patients with HER2-positive eBC [50] may help reduce this burden.

Our epidemiological projections should be considered within the context of therapeutic advancements in HER2-targeted therapies. Trastuzumab has led to substantial progress in reducing disease recurrence for patients with eBC [51]. For example, a recent model of United States patients diagnosed with HER2-positive eBC from 2006–2019 estimated that trastuzumab use was associated with 10,100 fewer distant recurrences compared with chemotherapy alone [52]. Our work builds on this by projecting the population-level benefit of T-DM1 in EU5 and Canada. The therapeutic landscape for HER2-positive cancer therapies continues to expand, with promising novel treatments under investigation for the population we studied. For example, the phase III DESTINY-Breast05 trial is evaluating trastuzumab deruxtecan vs T-DM1 in patients with high-risk HER2-positive BC with residual disease following neoadjuvant therapy (NCT04622319). The phase III ASTEFANIA study is evaluating the efficacy and safety of adjuvant T-DM1 with and without atezolizumab, an immunotherapy, in patients with residual disease following neoadjuvant therapy (NCT04873362). The phase III CompassHER2 RD trial is assessing the efficacy of T-DM1 with and without tucatinib in patients with high-risk HER2-positive BC (NCT04457596). Primary study completion dates are anticipated in 2025 for DESTINY-Breast05 and ASTEFANIA, and 2028 for CompassHER2 RD. These and other treatment options may provide further population-level benefit for our indicated populations in years coinciding with our study period. Improvements in neoadjuvant therapy may also benefit our indicated population, prior to assessment of residual disease. PHERGAIN-2 (NCT04733118) is a single-arm phase II study to assess efficacy of a chemotherapy-free pCR-guided strategy with trastuzumab and pertuzumab and T-DM1. APTneo is an enrolling phase III study of trastuzumab and pertuzumab with carboplatin and paclitaxel with and without atezolizumab among women with early high-risk and locally advanced HER2-positive suitable for neoadjuvant therapy (NCT03595592). Primary study completion dates are anticipated in 2025 for PHERGAIN-2 and 2026 for APTneo. Moreover, improvements in treatment options during the study period may contribute to further reductions in population-level mBC relapses in these settings.

Future work may elucidate the economic and other non-clinical implications of population-level improvements in relapsed mBC in EU5 and Canada. A 2014 Canadian study reported overall mean cost per case increases by stage during the first 2 years after diagnosis from a public payer perspective: I, $29,938; II, $46,893; III, $65,369; and IV, $66,627 [53]. Other studies in Italy, Spain, and the UK have also provided evidence of higher costs of treating mBC vs eBC in EU5 settings [54, 55]. At a population level, these costs appear to be considerable and increasing. In the US, total medical and productivity costs of mBC are estimated to increase from $63.4 billion in 2015 to $152.4 billion in 2030 [56]. Furthermore, beyond monetary costs, patients with recurrent BC, particularly those with distant recurrence, are more likely to experience poorer quality of life than women free of recurrence [57]; including more cancer-related stress [57, 58], increased use of anti-depressants [59], and less sexual intercourse [60]. Moreover, further work is necessary to inform how epidemiology prediction changes will contribute to changes in treatment-related costs, particularly for high costs of metastatic disease. Altogether, our results suggest that adjuvant T-DM1 treatment in EU5 and Canada may contribute to substantial population-level reductions in treatment-related costs associated with mBC.

Our study is subject to several limitations. Transporting data from a highly selected study population in a controlled interventional environment into the general population not in a controlled interventional environment constitutes a major study limitation. This limitation provides a threat to the validity of our estimates for subgroups not well-represented in KATHERINE since we were unable to account for relevant differences between trial and target populations. Future work using real-world data could help to inform the potential transportability of data from KATHERINE into unselected, uncontrolled real-world settings in EU5 and Canada. Projections from the current study are also limited by the shortcomings inherent to prediction models. First, the model estimates may not consider shifts in disease incidence over time or reflect actual treatment practice or changes that occurred after the model was developed. For example, results in Scenario 6 demonstrated that findings are highly sensitive to real-world treatment uptake, and we did not account for treatment discontinuation or lack of adherence to the labels. Results in Scenarios 7, 8 and 9 underscored that model projections were also highly sensitive to changes in neoadjuvant treatment preceding T-DM1 eligibility. In Scenario 9 we accounted for the expectation of increases in neoadjuvant treatment after 2020 following regulatory approvals in Canada, indicating that our main findings for Canada may be considered conservative in this regard. Second, while the use of real-world data might be considered a preferred source, pCR rates used to inform the base model were derived from the randomized, phase III NeoSphere study. The pCR rates from NeoSphere were lower than those reported from observational studies, as identified in a systematic literature review (data on file) conducted as part of our sensitivity analysis in Scenario 5. Use of lower pCR rates in the base model resulted in larger proportions of patients with HER2-positive eBC being eligible for adjuvant T-DM1 over 2021–2030. In comparison, while smaller proportions of patients with HER2-positive eBC were eligible for adjuvant T-DM1 in our sensitivity analyses using high pCR rates (Scenario 5), reductions in the numbers of cases of relapsed mBC could still be anticipated. By only using data from NeoSphere, for our base model, we ensured that inter- and intra-study variability in pCR rates following neoadjuvant trastuzumab in subgroups of patients with HR-positive vs HR-negative disease were minimized. While the use of data from a clinical trial setting has limitations, when comparing the results of the base model with those from Scenario 5 of the sensitivity analyses, which were informed by observational studies and the KRISTINE trial [49], it must be noted that the observational studies identified in the systematic literature review were of varying methodological quality and that clinical trials and observational studies recruit different patient populations. Thus, although outcomes from the two study types may differ and the magnitude of this difference is unknown, between our base case and Scenario 5 we have accounted for the potential impact of a range of pCR rates in the neoadjuvant setting. Third, our base model assumed that all neoadjuvant treatment was with trastuzumab rather than trastuzumab plus pertuzumab, which may result in an overestimation of the number of patients eligible for adjuvant T-DM1 treatment and, thus, an overestimation of the impact of adjuvant T-DM1. Neoadjuvant pertuzumab use was not included in the base model, as this would introduce uncertainties into the model concerning continued usage of pertuzumab in the adjuvant residual disease setting, and our intent was to focus on understanding the impact of adjuvant trastuzumab versus T-DM1. However, the pCR rates used in Scenario 5 assumed a 100% uptake of pertuzumab. Owing to limited available data, the model also did not consider the fact that patients with node-positive disease who did not have a pCR may benefit from concomitant treatment with trastuzumab plus pertuzumab vs trastuzumab alone. Additionally, our models did not consider vulnerable sub-populations with suboptimal treatment adherence. For example, a recent study of elderly populations in the US demonstrated substantial disparities in trastuzumab utilization [61], underscoring the importance of equitable access to medicines to ensure that future population-level improvements are experienced optimally across population groups.

Because the model outputs were dependent on extrapolated iDFS curves from KATHERINE, sensitivity analyses using different extrapolation assumptions were undertaken (i.e. exponential distribution, not log-normal; scenario 3). Our sensitivity analyses did not show major differences compared with the base models, providing confidence in the robustness of the base models. Accuracy of these epidemiology models will increase as more data from KATHERINE become available. An additional limitation of our models is that they did not account for the effects of the COVID-19 pandemic on patient care including delays in BC screening, diagnosis, and treatment. This could have impacted mortality assumptions in the model, as each 4-week delay in initiating the adjuvant treatment of BC is associated with a 9% increase in the risk of death [62]. Another limitation of the present analyses is that the incidence of relapsed mBC was determined using an extrapolated iDFS curve that included not only distant recurrence but also non-metastatic (local) recurrences. Although a split in type of recurrence was made based on the KATHERINE trial [19], as a simplification the rate in metastatic vs local recurrence was assumed to be constant over the 10-year period that the iDFS curves was extrapolated. This may be a limitation as the rate of metastatic vs local recurrence may change over the disease period. However, any change in the type of recurrence would affect both treatment options (trastuzumab and T-DM1); thus, the impact of this simplification is not expected to be considerable. Amidst these limitations, a strength of our analysis is the choice of “relapse to metastatic breast cancer” as an endpoint, rather than iDFS. While overall survival is the preferred endpoint for any clinical trial in cancer, iDFS is usually used as a surrogate endpoint. We, instead, extrapolated metastatic recurrence from the KATHERINE study. This allowed us to focus on metastasis-free survival as a clinically relevant endpoint that accounts for the availability of curative, local treatment options for locoregional occurrence and offers a meaningful measure of impact on an individual- and population-level. In addition, our methodology for extrapolating iDFS data is based on prior work by Sussell et al. [40], which provides a starting point for the complex epidemiological modeling presented herein. Collectively, our modeling results may inform future clinical and real-world data studies of T-DM1 and other HER2-targeted therapies in patients with HER2-positive eBC.

## Conclusion

Our results suggest that T-DM1 utilization in the EU5 and Canada has the potential to change the epidemiology of HER2-positive BC by preventing a substantial number of women with eBC and residual disease following neoadjuvant therapy from relapsing to mBC. These treatment effects may translate to declines in the clinical and economic burden of HER2-positive BC, particularly among women at an increased risk of disease recurrence. This work may help to inform future clinical and financial decision-making by healthcare systems.

### Supplementary Information

Below is the link to the electronic supplementary material.

Supplementary file1 (PDF 112 kb)

## Data Availability

For up-to-date details on Roche's Global Policy on the Sharing of Clinical Information and how to request access to related clinical study documents, see here: https://go.roche.com/data_sharing. The data that support the findings of this study are available from the corresponding author upon reasonable request from qualified researchers. Anonymized records for individual patients across more than one data source external to Roche cannot, and should not, be linked due to a potential increase in risk of patient re-identification.

## Abbreviations

BC: Breast cancer
COVID-19: Coronavirus disease 2019
eBC: Early-stage breast cancer
EMA: European Medicines Agency
EU5: 5 European countries (France, Germany, Italy, Spain, United Kingdom)
HER2: Human epidermal growth factor receptor 2
HR: Hormone receptor
iDFS: Invasive disease-free survival
mBC: Metastatic breast cancer
OS: Overall survival
pCR: Pathologic complete response
T-DM1: Trastuzumab emtansine
