# Supplementary material for: Population-level impact of adjuvant trastuzumab emtansine on the incidence of metastatic breast cancer: an epidemiological prediction model of women with HER2-positive early breast cancer and residual disease following neoadjuvant therapy
Source: Breast Cancer. 2023 Nov 1;31(1):84–95. doi: 10.1007/s12282-023-01514-w (PMC10764576; doi:10.1007/s12282-023-01514-w)
Supplement: Supplementary file 1 — Supplementary file1 (PDF 112 kb) [file 12282_2023_1514_MOESM1_ESM.pdf]

Supplemental Appendix

Supplemental Methods: Description of Sensitivity Analyses

Sensitivity analyses of five scenarios under different assumptions were performed to assess the magnitude of the change relative to the base model in the number of women projected to experience BC relapse following receipt of adjuvant T-DM1 vs. adjuvant trastuzumab. Scenario 1 assumed that cure is not possible. Scenario 2 assumed that the incremental treatment effect of T-DM1 over trastuzumab stopped at Month 63, the maximum follow-up duration for iDFS in the KATHERINE study (NCT01772472) [1] at the primary iDFS analysis. Scenario 3 assumed that the iDFS extrapolation distribution was exponential (i.e., not log-normal). Scenario 4 combines the above assumptions (i.e., Scenarios 1 to 3). Scenario 5 was based on a sensitivity analysis that considered different neoadjuvant treatments (pertuzumab) and rates of pCR. For this analysis, we conducted a systematic literature review to identify pCR rates following neoadjuvant treatment with trastuzumab in observational studies. We also considered pCR rates from applicable randomized controlled studies. For scenario 5, we report the upper limit of this sensitivity analysis based on the highest pCR rates identified, which came from the docetaxel, carboplatin, and trastuzumab plus pertuzumab group of the KRISTINE study (NCT02131064) [2]. Specifically, the KRISTINE study reported pCR rates of 43.8% among patients with HR-positive disease and 73.2% in those with HR-negative disease [2]. Scenario 6 assumed a peak 100% treatment share with T-DM1. Scenarios 7 and 8 assumed a 25% relative increase or decrease, respectively, in the proportion of women receiving neoadjuvant treatment. For Scenario 9 in the Canadian population, we also assumed a doubling of neoadjuvant treatment for years 2020 onwards (i.e., 40%), to account for clinical practice changes following drug approvals in Canada.

Supplemental References

1. von Minckwitz G, Huang CS, Mano MS, Loibl S, Mamounas EP, Untch M et al (2019) Trastuzumab emtansine for residual invasive HER2-positive breast cancer. N Engl J Med 380(7):617–628
2. Hurvitz SA, Martin M, Symmans WF, Jung KH, Huang CS, Thompson AM et al (2018) Neoadjuvant trastuzumab, pertuzumab, and chemotherapy vs trastuzumab emtansine plus pertuzumab in patients with HER2-positive breast cancer (KRISTINE): a randomised, open-label, multicentre, phase 3 trial. Lancet Oncol 19(1):115–126

Supplemental Table 1. Women projected to experience breast cancer relapse by geographic setting, adjuvant treatment regimen (T-DM1 or trastuzumab) and time period, among women diagnosed with HER2-positive eBC who have residual disease following neoadjuvant therapy from 2021 to 2030: sensitivity analyses

Analytical scenarios	Women projected to experience BC relapse in calendar year 2030		
	Following trastuzumab (n)	Following T-DM1 (n)	Difference across scenarios (%)
**Five European countries (EU5)[a]**			
Base model	3,691	2,460	(Reference)
1) Assumed cure is not possible	4,533	3,192	8.2%
2) Assumed the incremental treatment effect of T-DM1 over trastuzumab stops at 63 months (maximum iDFS follow-up from KATHERINE[b])	3,691	2,682	−22.0%
3) Assumes iDFS extrapolation distribution is exponential (not log-normal)	4,132	2,796	7.9%
4) Combines the above assumptions	5,392	4,547	−45.7%
5) Assumed high pCR rates from KRISTINE[c]	2,492	1,661	−48.1%
6) Assumed a peak 100% treatment proportion for T-DM1	3,691	2,152	20.0%
7) Assumed a 25% relative increase in neoadjuvant treatment for all years	4,735	3,156	22.0%
8) Assumed a 25% relative decrease in neoadjuvant treatment for all years	2,841	1,893	−29.9%
**Canada**			
Base model	249	166	(Reference)
1) Assumed cure is not possible	304	213	8.8%
2) Assumed the incremental treatment effect of T-DM1 over trastuzumab stops at 63 months (maximum iDFS follow-up from KATHERINE[b])	249	180	−20.3%
3) Assumes iDFS extrapolation distribution is exponential (not log-normal)	278	187	8.8%
4) Combines the above assumptions	359	301	−43.1%
5) Assumes high pCR rates from KRISTINE[c]	158	105	−56.6%
6) Assumed a peak 100% treatment proportion for T-DM1	249	145	20.2%%
7) Assumed a 25% relative increase in neoadjuvant treatment for all years	311	207	20.2%
8) Assumed a 25% relative decrease in neoadjuvant treatment for all years	187	124	-31.7%
9) Assumed a doubling of neoadjuvant treatment for years 2020 onwards	498	331	50.3%

BC breast cancer; *eBC* early stage breast cancer; *EU5* five European countries; *HER2* human epidermal receptor growth factor 2; *HR* hormone receptor; *iDFS* invasive disease-free survival; *pCR* pathological complete response; *T-DM1* trastuzumab emtansine

[a]Includes Germany, Spain, Italy, the United Kingdom, and France

[b]KATHERINE ClinicalTrials.gov number, NCT01772472

[c]For the pCR rate sensitivity analysis, we conducted a systematic literature review to identify pCR rates following neoadjuvant treatment with trastuzumab in observational studies and also considered pCR rates from applicable randomized controlled studies, all of which were used in the sensitivity analysis. Here we report the upper limit of this sensitivity analysis, based on the highest pCR rates identified. These came from the docetaxel, carboplatin, and trastuzumab plus pertuzumab group of the KRISTINE study, which reported a pCR rate of 43.8% in those with HR-positive disease and 73.2% in those with HR-negative disease [Hurvitz SA, et al. Lancet Oncol. 2018;19(1):115–26]. KRISTINE ClinicalTrials.gov number, NCT02131064.

Supplemental Figure 1. Model of iDFS for adjuvant T-DM1 vs. adjuvant trastuzumab[a]

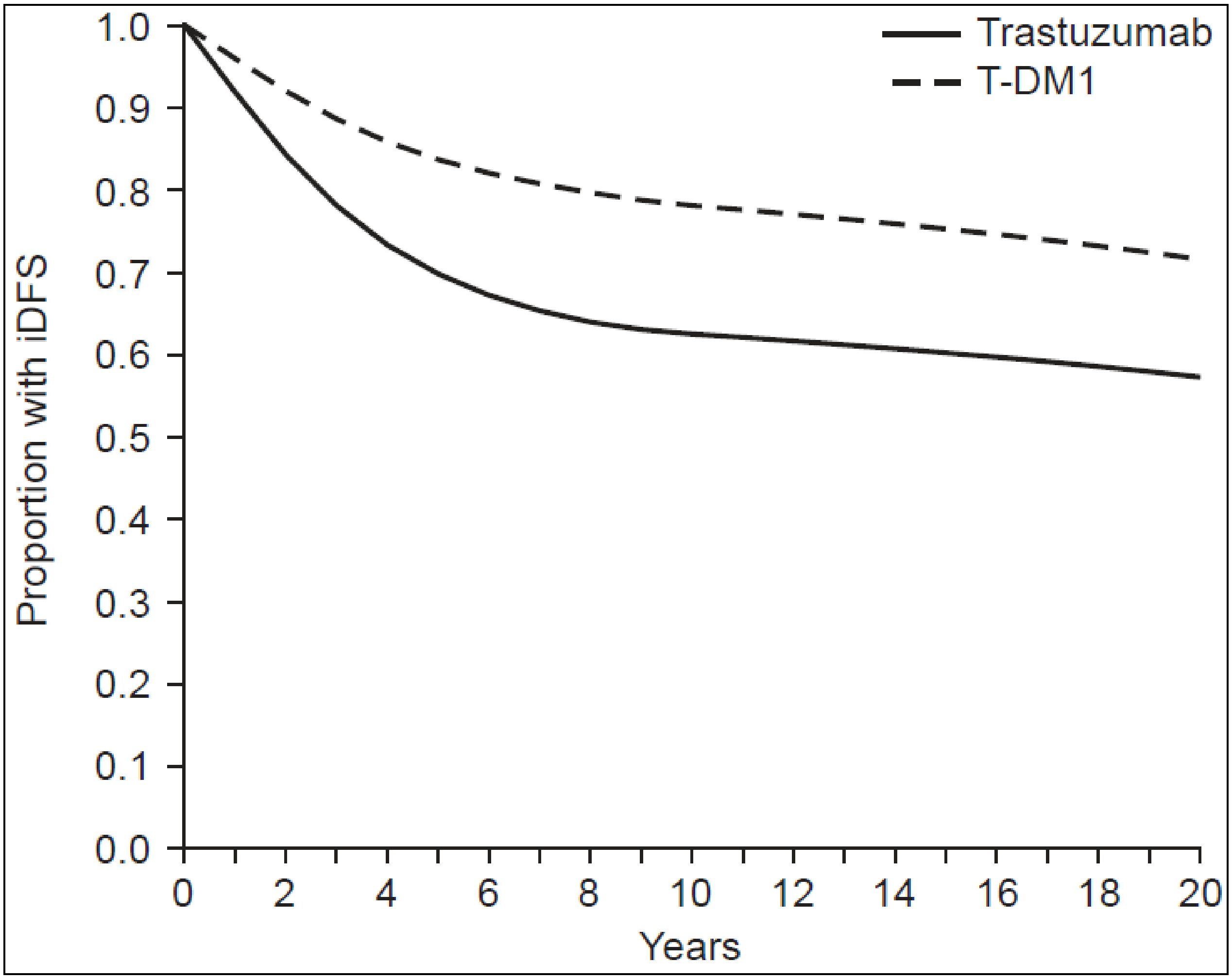

eBC early breast cancer; *HER2* human epidermal growth factor receptor 2; *iDFS* invasive disease-free survival; *T-DM1* trastuzumab emtansine

[a]Based on data extrapolated from KATHERINE trial, using a log-normal distribution, adjusted with cure and duration of treatment effect assumptions

Trastuzumab data were extrapolated from the trastuzumab arm of the KATHERINE study and were modeled beginning in 2006, the year trastuzumab was approved as an adjuvant treatment. T-DM1 data were extrapolated from the T-DM1 arm of the KATHERINE study and were modeled beginning in 2020, the launch of adjuvant use of T-DM1 in patients with HER2-positive eBC and residual disease following neoadjuvant treatment
